# Time-efficient germ cell transplantation from goldfish (*Carassius auratus*) into adult common carp (*Cyprinus carpio*)

**DOI:** 10.1590/1984-3143-AR2023-0121

**Published:** 2024-02-12

**Authors:** Angel Andreas Arias Vigoya, Daniel Fernandes da Costa, Marcos Antônio de Oliveira, Arno Juliano Butzge, Ivana Felipe Rosa, Lucas Benites Doretto, Emanuel Ricardo Monteiro Martinez, Melanie Digmayer, Rafael Henrique Nóbrega

**Affiliations:** 1 Centro de Aquicultura, Universidade Estadual Paulista, Jaboticabal, SP, Brasil; 2 Facultad de Medicina Veterinaria y Zootecnia, Fundación Universitaria San Martín, Bogotá, Colombia; 3 Departamento de Biologia Estrutural e Funcional, Instituto de Biosciências, Universidade Estadual Paulista, Botucatu, SP, Brasil; 4 National Key Laboratory of Mariculture Biobreeding and Sustainable Goods, Yellow Sea Fisheries Research Institute, Chinese Academy of Fishery Sciences, Qingdao, China

**Keywords:** biotechnology, spermatogonial stem cells, spermatogenesis, teleost fish, goldfish, common carp

## Abstract

Germ cell transplantation in fish is a promising technique for surrogate broodstock parents with broader application in aquaculture and conserving endangered and valuable genetic resources. Herein, we describe the establishment of an intrapapillary xenogeneic transplant of germ cells from sexually mature goldfish (*C. auratus*) males into common carp (*C. carpio*) males cytoablated with a thermochemical treatment (two doses of busulfan at 40 mg/kg at 35°C). To analyze the presence and development of donor germ cells in recipient testes, donor germ cells were labeled with PKH26, a fluorescent cell membrane dye, before transplantation. Our results demonstrated that thermochemical treatment caused effective spermatogenesis suppression and pronounced germ cell loss. Moreover, transplanted spermatogonial cells were able to colonize the recipients’ testes, resume spermatogenesis, and generate spermatozoa within eight weeks after germ cell transplantation. These findings suggested that recipient testes provided suitable conditions for the survival, colonization, proliferation, and differentiation of donor spermatogonia from a related species. This study indicated that recipients’ testes exhibited a high degree of plasticity to accept and support xenogeneic donor germ cells, which were able to form sperm in a short time frame. This approach has significant implications for assisted animal reproduction, biotechnology, conservation, and the production of valuable genetic resources and endangered fish species.

## Introduction

Spermatogonial stem cells (SSCs) play a pivotal role in maintaining sperm production in fish by either renewing themselves or differentiating into sperm-producing cells (Schulz et al., 2010; De Rooij, 2017, 2018; Xie et al., 2020). Due to the plasticity of SSC, transplantation techniques have been widely employed in managing aquatic resources, promoting genetic diversity, and enhancing the robustness of fish populations (Brinster and Avarbock, 1994; Nóbrega et al., 2010; Silva et al., 2016; Yoshizaki and Yazawa, 2019; Comizzoli and Holt, 2019; Liu, 2022; Tao and Hu, 2022; Yue and Shen, 2022; Zhou et al., 2023; Nayak et al., 2023).

A significant breakthrough in the transplantation technique was achieved by Lacerda et al. (2010), who developed a nonsurgical approach for germ cell transplantation in Nile tilapia (*Oreochromis niloticus*). This method involved injecting donor SSCs into the testes of adult recipients through the urogenital papilla and spermatic duct, resulting in the restoration of gametogenesis and the production of functional, donor-derived gametes (Majhi et al., 2009; Nóbrega et al., 2010; Majhi et al., 2014; Silva et al., 2016; Xu et al., 2019).

Moreover, the utilization of adult recipients combined with this technique in SSC transplantation has proven to accelerate sperm production, offering a practical approach to accelerate the generation time of donor species (Lacerda et al., 2010; Xu et al., 2019). For instance, the efficient production of gametes in sexually mature fish has been notably demonstrated in tilapia (*Oreochromis niloticus*) and blue drum (*Nibea mitsukurii*). In the case of tilapia, spermatozoa production was observed seven weeks post-transplantation (Lacerda et al., 2010), while in blue drum, this production was observed nine weeks post-transplantation (Xu et al., 2019). Therefore, these findings underscore the time-efficient and successful production of gametes through nonsurgical transplantation, offering valuable insights into reproductive strategies in aquaculture.

In addition, the efficient establishment of donor germ cell gametogenesis in recipient testes relies on the availability of suitable niches for germline stem cells (Ogawa et al., 2000; Schulz and Miura, 2002). In this regard, strategies such as thermochemical treatments combining busulfan and high temperatures have been employed to eliminate host germ cells and suppress spermatogenesis in various adult fish species, including *Cyprinus carpio* (Vilela et al., 2003; Lacerda et al., 2013; Siqueira-Silva et al., 2021; Wang et al., 2021; Rosa et al., 2023; Vigoya et al., 2024).

Hence, considering the potential of nonsurgical SSC transplantation as a valuable tool for accelerating functional sperm production, the current study transplanted goldfish (*C. auratus*) SSCs into common carp (*C. carpio*) adult males previously depleted by busulfan and high-temperature treatments. ﻿

## Methods

### Experimental animals and rearing procedures

Sexually mature male common carp (n=20) and goldfish (n=5) were housed in 200-liter tanks maintained at 26°C and subjected to a natural photoperiod of 14 hours light and 10 hours of darkness. The animals were fed a pelleted commercial diet three times daily. The specimens were sourced from commercial breeders and raised in the aquarium facility located within the Department of Structural and Functional Biology at the Institute of Biosciences, São Paulo State University (Botucatu, Brazil). The study was approved by the National Council for the Control of Animal Experimentation (CONCEA) and the Ethics Committee on Animal Care and Experimentation of São Paulo State University (CEUA) under protocol n° 5229/15.

### Recipient preparation

In this study, we used a protocol that combines high water temperature with the cytostatic agent busulfan (Sigma‒Aldrich) to suppress the recipient's spermatogenesis (Lacerda et al., 2013). Before treatment, fish were subjected to an acclimation period of two weeks, where the water temperature was gradually increased until reaching 35°C. Subsequently, the animals received two intracoelomic busulfan injections (40 mg/kg/BW) dissolved in vehicle solution (dimethyl sulfoxide - DMSO diluted in phosphate-buffered saline solution - PBS), with a two-week interval between injections.

### Isolation of donor germ cells for transplantation

Donor germ cells from adult goldfish (*Carassius auratus*) were isolated based on the protocol described by Lacerda et al. (2010). Briefly, sexually mature males (n=5) were sacrificed by anesthetic overdose, and testes were excised, finely cut, and rinsed in Hank´s balanced salt solution (HBSS). Testicular tissue was finely minced and incubated in a dissociating solution containing 0.2% collagenase in DMEM/F12 medium for 3 h at 26°C and 80 rpm. The solution was filtered using a nylon membrane (mesh size 70 μm) to eliminate the undissociated cell clusters, centrifuged for 10 min at 1200 rpm, and resuspended in DMEM-BSA 0.75% medium. The solution was suspended in a discontinuous Percoll density gradient, from the higher (40%) to lower (15%) gradient and centrifuged at 1200 rpm for 30 min at 25°C. Bands enriched in spermatogonial stem cells (SSC) were collected, the cells were washed, and their viability was assessed through a Trypan blue (0.4%) exclusion assay.

### Light microscopy analysis

For morphological analyses of Percoll bands, cells were rinsed in DMEM-BSA 0.75%, centrifuged, and fixed in 5% buffered glutaraldehyde (0.05 M phosphate buffer, pH 7,3) for 24 h at 4°C. Posteriorly, the cells were dehydrated in a graded ethylic series and embedded in methyl methacrylate (Leica Microsystems). Then, the blocks were sectioned (3 μm thickness), stained with 1% toluidine blue, and analyzed using a Leica DMI 4000 B microscope. The cellular characterization of each Percoll layer was based on the morphological criteria described by Lacerda et al., 2019.

### Germ cell labeling and transplantation

Before transplantation, donor cells were labeled with the fluorescent membrane dye PKH26 (Sigma‒Aldrich, St. Louis, MO, USA) to track their localization and development inside the recipient testes. Germ cells were stained following the manufacturer’s guidelines at a concentration of 8 µL per mL for 5 min at room temperature. Then, labeled germ cells were rinsed three times with DMEM/F12, suspended in DMEM-BSA 0.75%, and stored on ice until transplantation. Thus, two weeks after the second busulfan injection, recipient common carp (n=20) were anesthetized with benzocaine (75 mg per L of water), and the donor germ cells were injected through the urogenital papilla using a micropipette (70 µm diameter) under a stereomicroscope (Zeiss 475052, Germany). Each individual was injected with 1 mL of cell suspension containing DMEM and 0.4% trypan blue solution (1:10) at a concentration of approximately 1x10^7^ cells/mL. After transplantation, animals were reared in tanks in which water temperature was decreased 1-2°C per day until reaching 26°C.

### Germ cell transplantation analysis

To assess transplantation efficiency (colonization of recipient testes), the fate, and the post transplantation development (proliferation and differentiation) of donor germ cells, testes transplanted with PKH26-labeled germ cells were collected at specific periods, from one to 8 weeks. To this end, testes from transplanted animals (n=20) were removed, embedded in Jung Tissue Freezing Medium (Leica Instruments, Nussloch, Germany), frozen in liquid nitrogen, and stored at -80°C until sectioning. Tissue samples were sectioned at a thickness of 10 µm through a cryostat (Leica CM 1850, Germany) and stained with DAPI (targeting DNA in the cell nucleus). The obtained sections were observed, analyzed, and photographed under a fluorescent Leica DMI 4000 B microscope.

### Ethics statement

All procedures were consistent with Brazilian national regulations and followed the guidelines for ethical animal treatment approved by the National Council for the Control of Animal Experimentation (CONCEA) and the Ethics Committee on Animal Care and Experimentation of São Paulo State University (CEUA), protocol n° 5229/15.

## Results

### Recipient preparation

**Figure 1:**
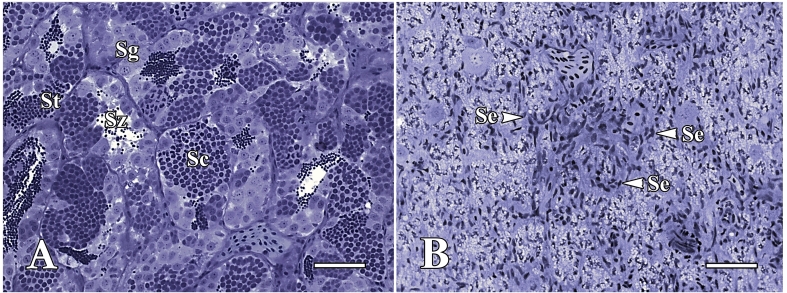
Histology of sexually mature common carp testis kept at 35°C, control (A), and treated with busulfan at high temperature, 35°C (B). The seminiferous tubules of control fish show spermatogenic cysts at different phases of development, whereas Sertoli cells-only (Se) are present in most seminiferous tubules of busulfan-treated common carp. Sertoli cell only (Se), spermatocytes (Sc), spermatogonia (Sg), spermatids (St), spermatozoa (Sz). Scale bars: 50 µm.

To deplete endogenous recipient spermatogenesis, we employed high-temperature and busulfan treatments. Our results revealed that the combined treatment effectively suppressed common carp spermatogenesis, as supported by histological analysis. In detail, acute atrophy of the germinal epithelium and the absence of spermatogenic cysts were observed two weeks post treatment. Moreover, the spermatogenic tubules displayed a Sertoli cell-only phenotype, while the control testes exhibited typical spermatogenesis progression with spermatocytes at varying stages (Figure 1.) In addition, all recipient common carp exhibited good tolerance, achieving a 100% survival rate without any external injuries or pathologies.

### Isolation of donor germ cells for transplantation

**Figure 2:**
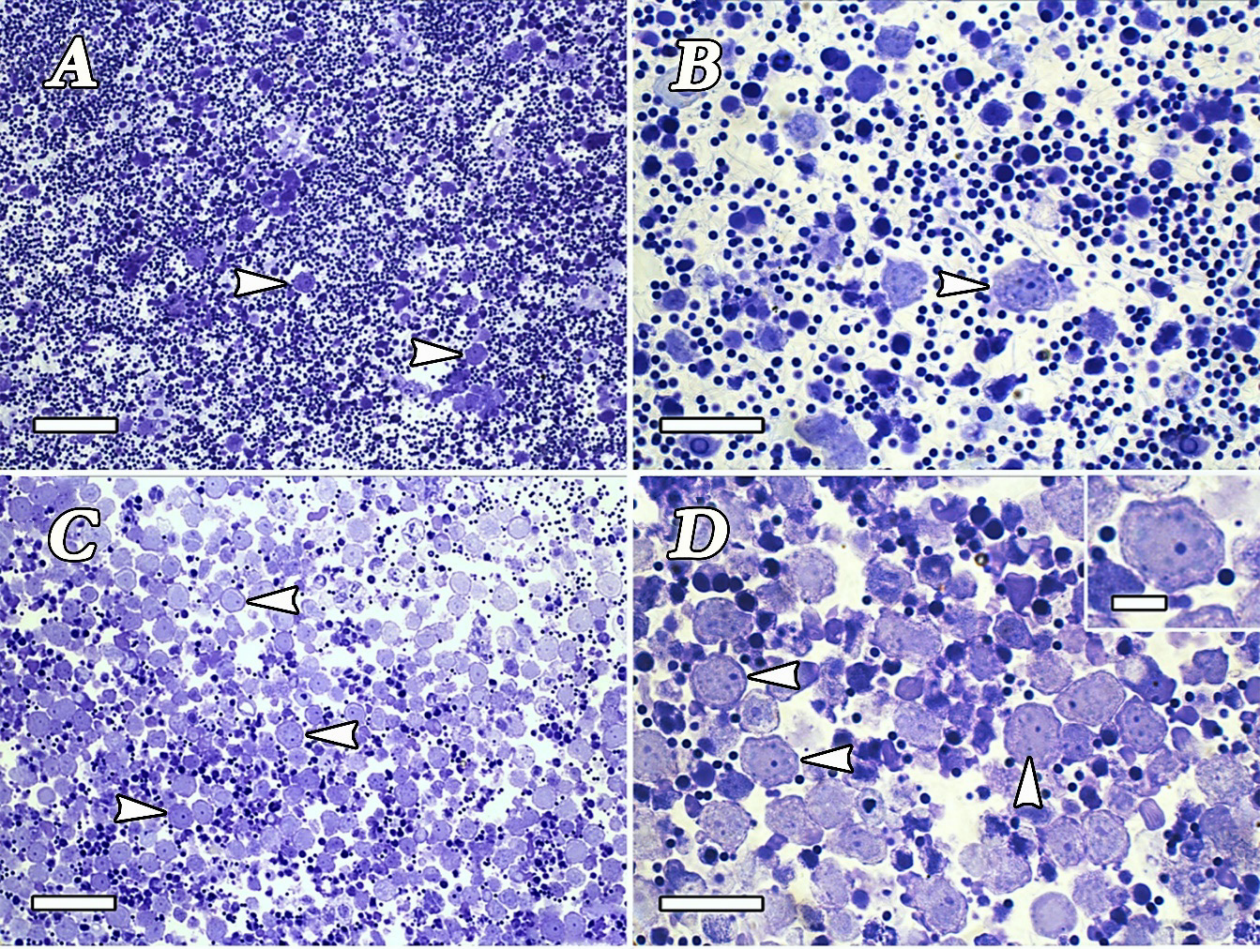
Heterogeneous donor germ cell suspension before Percoll gradient centrifugation. A mixture of different germ cell types and somatic cells formed this suspension (A-B). After density gradient centrifugation (Percoll), a donor germ cell suspension enriched with type A undifferentiated spermatogonia (arrowheads) was obtained. However, spermatocytes, spermatids, and spermatozoa were also observed (C-D). The inset in (D) shows a presumptive spermatogonial stem cell. Scale bars: A and C = 50 µm; B and D = 25 µm. Inset in D = 10 µm.

To isolate and enrich the SSC fraction used for transplantation, a protocol developed by Lacerda et al. (2013) was employed. This protocol allowed successful removal of somatic and mature germ cells and enrichment of SSCs. After enzymatic digestion, a heterogeneous cell suspension was submitted to a discontinuous Percoll density gradient. Six bands between Percoll interfaces were found. Spermatogonia enrichment was found in the upper Percoll bands, whereas mature spermatids, spermatozoa, and cell debris were pelleted down at the bottom of the tube. Further morphological analysis confirmed that the second and third layers contained a higher concentration of viable undifferentiated spermatogonia, including putative SSCs (Figure 2). These bands were selected for transplantation, and the cell concentration present in these two bands was estimated at approximately 1x10^7^ cells.

### Germ cell labeling and transplantation

**Figure 3:**
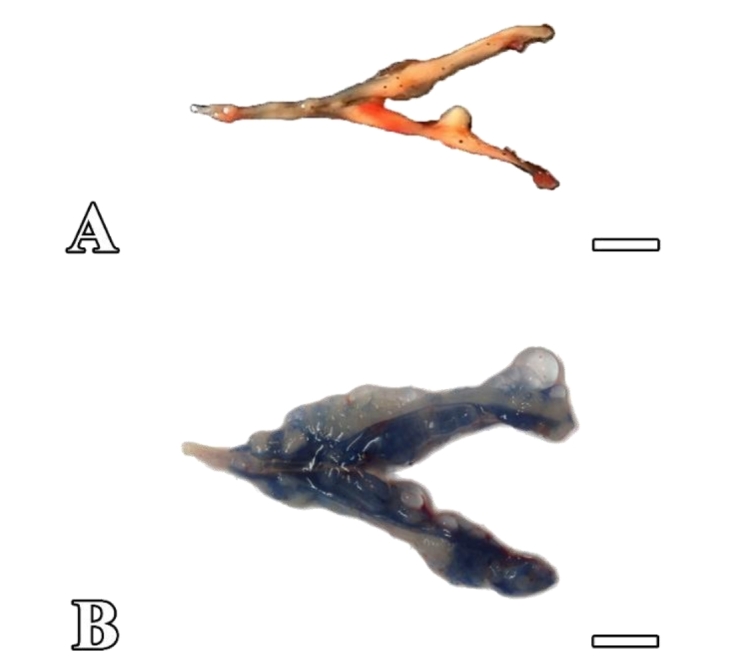
Control and transplanted testes of sexually mature common carp. (A) The appearance of the control testes treated with busulfan at 35°C, which did not receive germ cell transplantation. (B) Testes of recipients filled with the solution after transplantation. Scale bars: 1 cm.

In the present study, an enriched donor SSC suspension was transplanted into the receptor testes using a micropipette (nonsurgical procedure) through the urogenital papilla and the spermatic duct of common carp males. To assess the transplantation efficiency, a trypan blue solution was added to the cell suspension. After transplantation, recipient testes were labeled with trypan blue solution, indicating that the cell suspension was completely incorporated into the seminiferous tubules (Figure 3).

**Figure 4:**
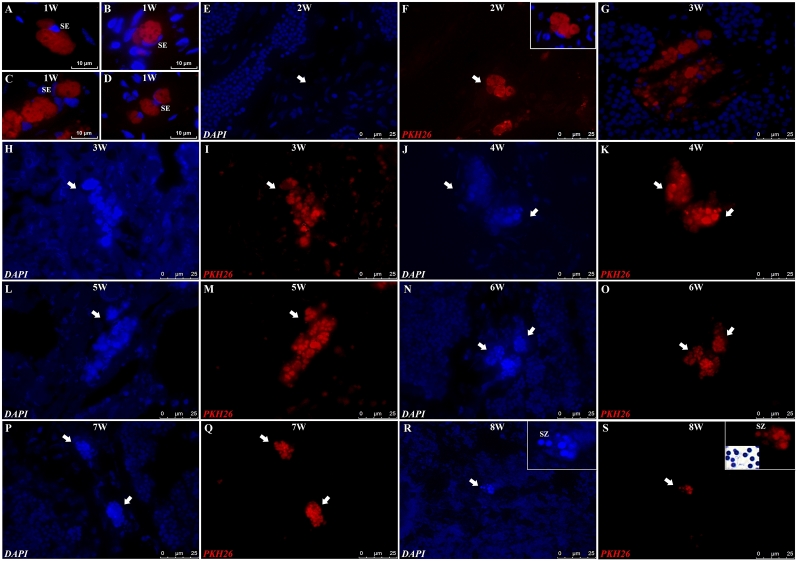
Microscopic evaluation of recipient common carp testis 1 to 8 weeks after goldfish spermatogonial cell transplantation. The post transplantation interval (in weeks) is shown at the top of each figure. At one week (A-D) post transplantation, PKH26-labeled spermatogonial cells (labeled in red) were observed in the common carp seminiferous tubule epithelium. From two to eight weeks after transplantation (E-S), these PKH26-labeled germ cells (in red), also labeled with DAPI (cell nuclei labeled in blue; E, H, J, L, N, P, R), formed spermatogenic cysts of different sizes. The insert in (F) represents the merged image from “F”. Eight weeks after transplantation, donor-derived spermatozoa labeled with DAPI (in blue) and labeled with PKH26 (in red) were observed in the recipient seminiferous tubule lumen. SZ: spermatozoa, SE: Sertoli cell.

The analysis of recipient testes demonstrated that one week after transplantation, donor PKH26-labeled germ cells (presumably SSCs) were distributed in the lumen of seminiferous tubules (Figure 4A, 4B, 4C, 4D). After two weeks, the transplanted cells migrated, colonized, and were able to form clusters in the recipient germinal epithelium (Figure 4E, 4F). Donor germ cells then proliferated and were organized in a cystic arrangement. PKH26-labeled cells forming spermatocytes of different sizes and at different developmental stages were observed in the recipient testes (Figure 4E, 4F). Three to seven weeks after transplantation, cysts increased in size and number (Figure 4G-4Q), and eight weeks post transplantation, cysts in advanced stages of development and presumably spermatids and spermatozoa were found (Figure 4R, 4S).

## Discussion

The transplantation of spermatogonial stem cells (SSCs) is a highly promising strategy to conserve economically important fish populations, mitigate reproductive barriers and enhance productivity in aquaculture (Hattori et al., 2019; Liu, 2022; Tao and Hu, 2022; Yue and Shen, 2022; Saleedang et al., 2022; Ryu et al., 2022; Zhou et al., 2023; Nayak et al., 2023).

Hence, taking into consideration that nonsurgical SSC transplantation into adult recipients represents an efficient approach to accelerate the generation time of donor fish species (Lacerda et al., 2010), the current study focused on achieving the transplantation of goldfish (*C. auratus*) SSCs into common carp (*C. carpio*) adult males, previously depleted by combining both busulfan and high-temperature treatments. ﻿

In alignment with our prior studies (Vigoya et al., 2024; Rosa et al., 2023), the combination treatment with busulfan and high temperature effectively suppressed the endogenous spermatogenesis of common carp males, resulting in seminiferous tubules with a Sertoli cell phenotype. Thereafter, we successfully transplanted goldfish germ cells into adult male common carp. Approximately eight weeks post-transplantation, PKH26-labeled spermatozoa were observed in recipient testes, indicating successful colonization, proliferation, and differentiation of donor-derived germ cells. In this sense, our findings indicate that common carp testes remain sexually competent and are able to support gametogenesis.

Remarkably, the time-efficient production of gametes using sexually mature fish is consistent with studies in tilapia (*Oreochromis niloticus*) (Lacerda et al., 2010) and in marine blue drum (*Nibea mitsukurii*) (Xu et al., 2019). For instance, in tilapia, functional sperm were formed within 9 weeks, while drum recipients showed the earliest spermatozoa production at 7 weeks post-transplantation (Lacerda et al., 2010; Xu et al., 2019). Furthermore, to our knowledge, our current study is the first to report goldfish spermatozoa production within a brief 8-week period post-transplantation, which is notably faster than the goldfish gamete production timeline (2 months) observed in the study conducted by Majhi (2023). Therefore, the rapid proliferation and differentiation of donor cells observed in previous studies may be attributed to the favorable microenvironment provided by the adult common carp testes. This microenvironment is commonly facilitated by the presence of recipient Sertoli cells, recognized for their pivotal role in establishing an immune-privileged environment within the testis (Fijak and Meinhardt, 2006; Halley et al., 2010; Sharma et al., 2022; Fujihara et al., 2022). In this context, the Sertoli cells in the recipient gonad may offer support to stained donor germ cells, promoting the development of new cysts. Furthermore, the improved effectiveness of our transplantation approach may also be influenced by shared physiological, reproductive, and phylogenetic characteristics among the selected species (Taylor and Mahon, 1977; Brzuska, 2014; Wang et al., 2014). Thus, the established transplantation system in this study has considerable advantages for the rapid breeding of farmed fish.

In addition to SSC transplantation into adult recipients, microinjection of primordial germ cells or spermatogonia into embryos or the peritoneal cavity of newly hatched larvae is an alternative method for the production of functional, donor-derived gametes (Saito et al., 2008; Takeuchi et al., 2001; Takeuchi et al., 2003; Takeuchi et al., 2009; Yoshizaki and Lee, 2018; Lee et al., 2021; Franěk et al., 2021; Saleedang et al., 2022). However, these methods require prolonged cultivation of the recipient animals before the recipient reaches sexual maturity and produces donor-derived functional gametes. Consequently, the techniques outlined in our study hold promise for broad applicability across a diverse range of fish species, overcoming the limitations previously mentioned.

## Conclusion

**Figure 5:**
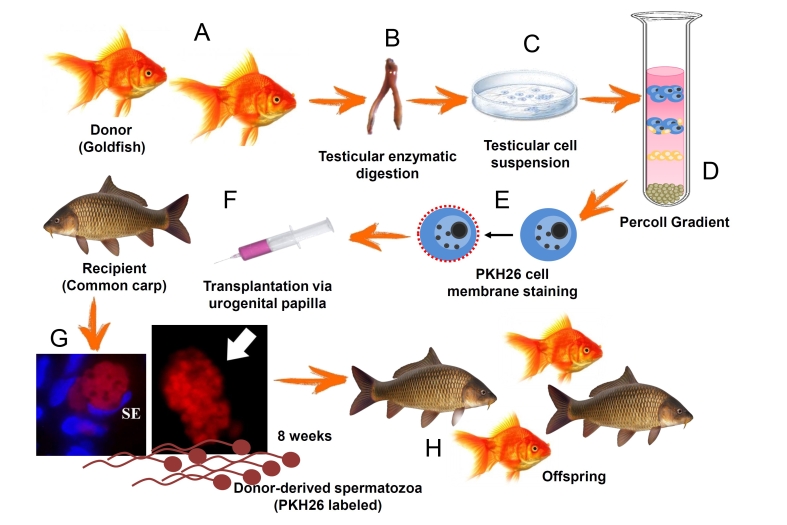
Summary of methodology and results obtained in the current study a﻿daptaded from Lacerda et al. (2010). Testicular tissue from goldfish was collected and subjected to enzymatic digestion to obtain a suspension of testicular cells (A, B, C). Prior to transplantation, donor cells enriched with Percoll were labeled with a fluorescent membrane dye, PKH26, to track their localization and development within the recipient testes (D, E, F). Ultimately, germ cells from goldfish successfully colonized, proliferated, and differentiated within the recipient gonads of common carp, leading to the production of gametes 8 weeks post transplantation (G) and offspring production (H). SE: Sértoli cell.

In conclusion, our study achieved the successful transplantation of goldfish germ cells into adult male common carp, leading to rapid sperm production (Figure 5). Thus, our data highlight the remarkable plasticity of common carp testes to accept and support goldfish germ cells. Therefore, this approach has significant implications for the rapid breeding of farmed fish, and for the conservation of valuable genetic resources.
